# The judicialization of the medical act^☆^

**DOI:** 10.1016/j.bjorl.2015.12.002

**Published:** 2015-12-13

**Authors:** Roberto Augusto de Carvalho Campos, Rosmari Aparecida Elias Camargo, Luciano Rodrigues Neves

**Affiliations:** a Escola Paulista de Medicina, Universidade Federal de São Paulo (UNIFESP), São Paulo, SP, Brazil; b Department of Otorhinolaryngology and Head and Neck Surgery, Universidade Federal de São Paulo (UNIFESP), São Paulo, SP, Brazil; c Department of Criminal Law, Forensic Medicine and Criminology, Faculdade de Direito, Universidade de São Paulo (USP), São Paulo, SP, Brazil

Since its beginning and, more effectively, since the Middle Ages, medical practice has been the subject of relentless social control and, consequently, of the Law itself. In this sense, Medieval Medicine was subjected to the practical analysis of obtained results, whether with the use of medications or surgical interventions, thus defining what therapeutic success, accidental result, unforeseeable circumstances or inexorable course of the disease were.^1^

From the XIX century on, there was an increase in at-risk surgical procedures and, simultaneously, there was an increase in patients’ complaints and their resistance to submit to the procedures indicated by physicians.^2^ It was observed that such events occurred precisely at a time of increasing investment in more effective surgical techniques and strategies that culminated in the decrease of hospital infection rates.

In the early XX century, due to the growth in legal complaints due to medical malpractice, there was ample discussion on the fact that the bad results that occurred should not be attributed exclusively to the surgeon or clinician who directly worked on the case. It is questioned, then, that the responsibility should be shared by other professionals who had the opportunity to act on or influence the assumed behaviors.

At the end of the XX century, medical malpractice became a public health problem, as the human and material resources used for the purpose of correction of “malpractice” increased considerably, amounting to approximately 100,000 cases/year in the United States.^3^

James Reason, a researcher at the University of Manchester, has discussed “medical malpractice” in several publications, and mainly recommencing the discussion that this malpractice could be systemic and organizational,^4^ intensifying the concern for a safer medical practice.

In Brazil, with the advent of the Consumer Protection Code and the considerable increase in demands for compensation based on alleged medical malpractice, the doctor–patient relationship gained a prominent place in the legal and academic circles and in the Courts.

Medical procedures have never been as formalized as they are today, with the prevailing objective of documenting the decisions and protect the interests, rights and duties of the parties involved, inexorably changing the doctor–patient relationship.

Some of these procedures that should be maintained in medical practice are described below:

- Drafting of the Free and Informed Consent (IFC) form;
- Photographic evidence or video recording of the pre- and postoperative status;
- Performance of the “check list” and “time out” in the Surgical Center and preparation of detailed reports whenever requested by the patient;
- Description in the medical records of the established diagnoses, as well as treatment options and their risks, always in the patient's presence after his or her consent;
- In all medical chart notes, accurately record the date of consultation and time of assessment, especially in case of hospitalization;
- Under no circumstances to comment about the case with personnel not committed to the ethical duty of confidentiality;
- In case of unexpected events, the physician should share such events with the patient, family or guardians, followed by information regarding alternative proposals for case management;
- Keep up to date with the Medical Ethics Code, Resolutions and Opinions issued by the Medical Council, to not practice proscribed procedures or those not yet scientifically approved;
- Whether a specialist or not, the physician should be updated as to the progress of Medicine, seeking to offer the best of the knowledge of diagnosis and treatment in favor of the patient.

Regardless of the strength of the said material evidence, according to our Law, one cannot interpret it alone or overestimate it, considering that it could avoid possible misunderstandings. Furthermore, there is a risk of transforming the doctor–patient relationship into a cool, formal contract, setting aside the dialog, attention and empathy, characteristics that guide the practice of medicine.

Another aspect to be considered refers to the so-called Defensive Medicine, i.e., the change in medical conduct from the usual behavior or that considered good medical practice to an attitude aiming to reduce or prevent questions or criticisms from their patients and relatives. Such practice can occur through two mechanisms – positive and negative. The first occurs when there is excessive test ordering or conducts for the treatment and the last by the withdrawn attitude on the part of the physician, by proposing referrals and taking evasive actions.

Is there a point of equilibrium for such situation?

We think there is one, in which physicians should always adequately document the care given to their patients, whereas they continue to advise them, using accessible, frank language, tailored to each patient, respecting their capacity to understand, favoring the autonomy of decision in true partnership, sharing the risks and possible poor outcomes.

## Conflicts of interest

The authors declare no conflicts of interest.
